# Pressure Control of Nonferroelastic Ferroelectric Domains in ErMnO_3_

**DOI:** 10.1021/acs.nanolett.3c01638

**Published:** 2023-07-20

**Authors:** Olav W. Sandvik, Aaron Merlin Müller, Håkon W. Ånes, Manuel Zahn, Jiali He, Manfred Fiebig, Thomas Lottermoser, Tadej Rojac, Dennis Meier, Jan Schultheiß

**Affiliations:** † Department of Materials Science and Engineering, Norwegian University of Science and Technology (NTNU), 7034 Trondheim, Norway; ‡ Department of Materials, ETH Zurich, 8093 Zurich, Switzerland; § Experimental Physics V, University of Augsburg, 86159 Augsburg, Germany; ∥ Electronic Ceramics Department, Jožef Stefan Institute, 1000 Ljubljana, Slovenia

**Keywords:** topologically protected defects, piezoresponse force microscopy, domain engineering, mechanical pressure, improper ferroelectrics

## Abstract

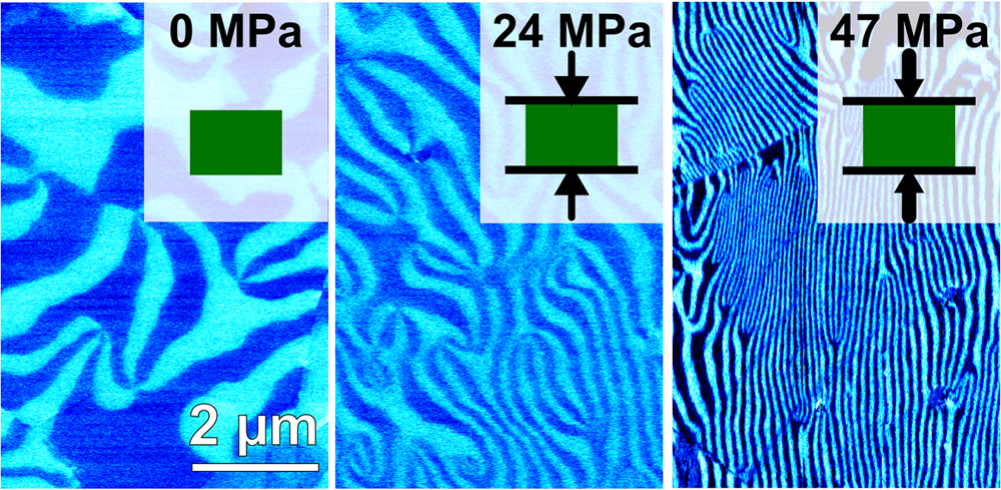

Mechanical pressure controls the structural, electric, and magnetic order in solid-state systems, allowing tailoring of their physical properties. A well-established example is ferroelastic ferroelectrics, where the coupling between pressure and the primary symmetry-breaking order parameter enables hysteretic switching of the strain state and ferroelectric domain engineering. Here, we study the pressure-driven response in a nonferroelastic ferroelectric, ErMnO_3_, where the classical stress–strain coupling is absent and the domain formation is governed by creation–annihilation processes of topological defects. By annealing ErMnO_3_ polycrystals under variable pressures in the MPa regime, we transform nonferroelastic vortex-like domains into stripe-like domains. The width of the stripe-like domains is determined by the applied pressure as we confirm by three-dimensional phase field simulations, showing that pressure leads to oriented layer-like periodic domains. Our work demonstrates the possibility to utilize mechanical pressure for domain engineering in nonferroelastic ferroelectrics, providing a lever to control their dielectric and piezoelectric responses.

The functionality of ferroelectrics is intimately coupled to their domain structure.^1^ This coupling allows for tuning the dielectric, piezoelectric, and electromechanical properties via domain engineering, which has been realized via the introduction of a morphotropic phase boundary,^2^ a critical point,^3^ or defect complexes.^4^ Another well-established and very versatile approach to property engineering is elastic strain, which has been applied to enhance the spontaneous polarization in BiFeO_3_ thin films,^5^ stabilize the ferroelectric order in SrTiO_3,_^6^ and tailor the domain density in BaTiO_3_ via extended defects^7^ or internal microstructural effects.^8^

In addition, the application of an external mechanical pressure has a substantial impact on the formation of domains, codetermining their size, shape, and stability and, hence, the macroscopic ferroelectric responses.^9−11^ Most existing concepts for ferroelectric domain engineering via elastic strain focus on the family of perovskite oxides, exploiting strain-induced ferroelastic domain walls that form in addition to the ferroelectric walls to release the strain.^12,13^ More recently, strain-driven domain engineering in nonferroelastic ferroelectrics, i.e., ferroelectrics that do not host ferroelastic domain walls, has drawn attention. In particular, improper ferroelectric hexagonal manganites (*R*MnO_3_, *R* = Sc, Y, In, and Dy–Lu),^14,15^ which are isostructural to hexagonal ferrites, indates, and gallates,^16,17^ have been studied. Ferroelectricity in *R*MnO_3_ arises as a secondary effect, driven by a trimerizing lattice distortion, which represents the primary symmetry-breaking order parameter.^18^ As a consequence, the ferroelectric domain structure of *R*MnO_3_ exhibits characteristic 6-fold meeting points of alternating ±*P* domains (*P* denotes the local polarization),^19^ forming topologically protected vortex/antivortex pairs. Related to the improper nature of the ferroelectricity, *R*MnO_3_ systems provide a large variety of unusual physical phenomena, ranging from charged domain walls with unique electronic properties^19−21^ to nonconventional domain-scaling behavior.^22−24^ The structural symmetry-breaking order parameter in *R*MnO_3_ is coelastic rather than ferroelastic, and all ferroelectric domain walls in the uniaxial system are of a 180° type. Importantly, this type of domain wall is not expected to move in response to an externally applied stress.^25^ Interestingly, domain structure engineering can be realized via a strain gradient. For the hexagonal manganites, an elastic strain gradient creates a pulling force on the vortex/antivortex pairs, resulting in a transformation of the isotropic vortex-like domains into stripe-like patterns^26^ and an inversion of the domain scaling behavior with respect to grain size compared to classical perovskite systems.^27^ These findings reflect completely different domain physics beyond what is known from ferroelastic ferroelectrics, representing a largely unexplored playground for the engineering of ferroelectric domains and polar nanostructures.

Here, we study the impact of uniaxial pressure up to the MPa regime on the domain formation in ErMnO_3_ polycrystals during high-temperature treatment through the Curie temperature. Our systematic analysis reveals a coherent response of the uniaxial ferroelectric grains, leading to a decrease in domain size with increasing pressure. Despite the random crystallographic orientation of the grains with respect to the direction of the applied pressure, we observe that all grains transform from an isotropic vortex-like domain structure to stripe-like domains as a function of the pressure, with the domain walls orienting parallel to the polar axis of each grain.

## Domain Morphology after High-Pressure Annealing

**Figure 1:**
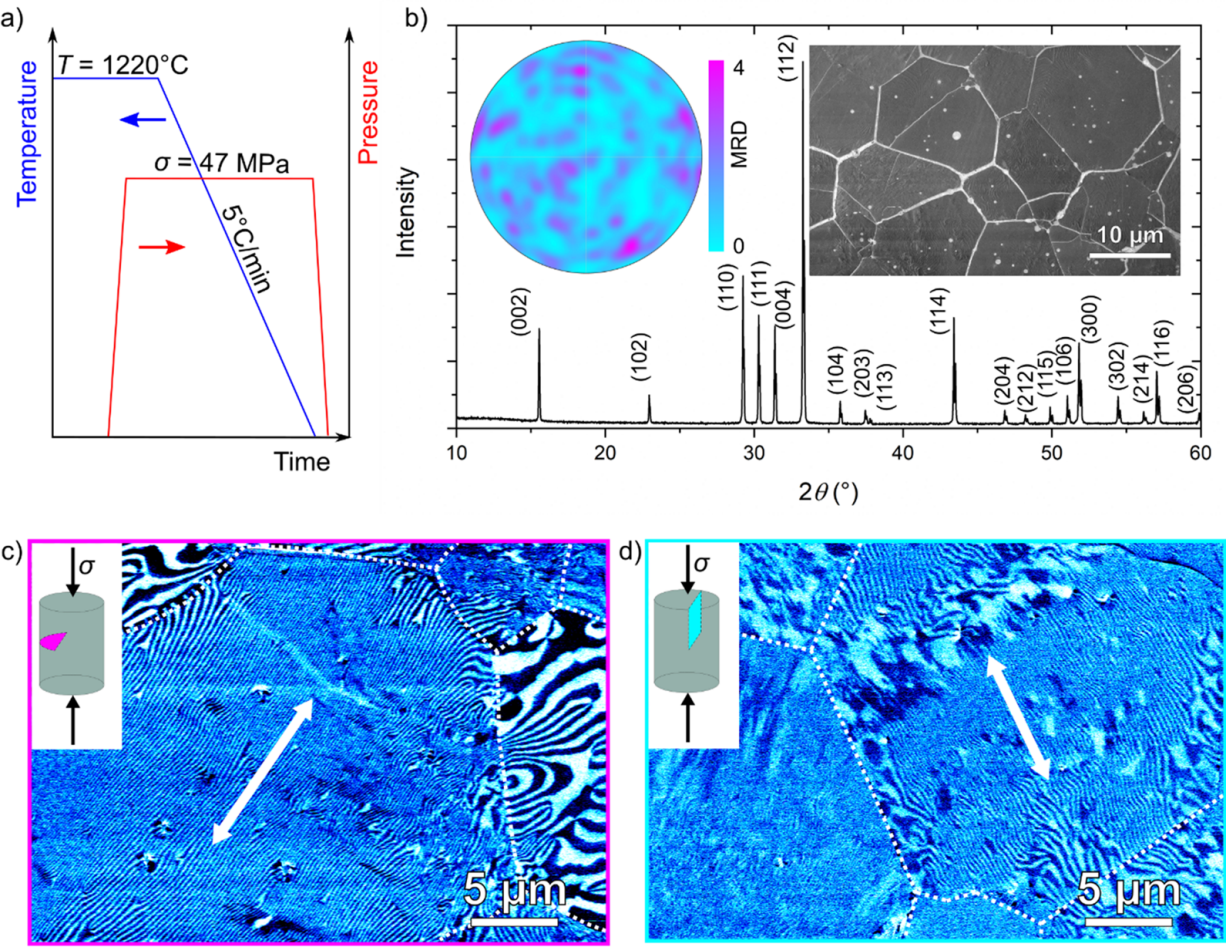
Ferroelectric domain structure in polycrystalline ErMnO_3_ cooled under applied mechanical pressure. a) Schematic temperature and pressure profile displaying the thermomechanical treatment of the sample from 1220 °C to room temperature under an applied uniaxial pressure of σ = 47 MPa. b) XRD pattern of ErMnO_3_ after annealing. Characteristic (*hkl*) reflections are marked. The pole figure in the inset, obtained from EBSD orientation data from ∼400 grains, displays a uniform distribution of the {1000} planes with multiples of a random distribution (MRD) value of <4 for all orientations. A micrograph of the sample obtained from SEM is displayed in the inset. In-plane PFM contrast displaying the domain structure of a cross section c) perpendicular and d) parallel to the applied pressure. The out-of-plane PFM contrast is displayed in Figure S1. The grain boundaries are indicated as dotted white lines. The preferential orientation of the stripe-like domains within one grain along the white arrows is highlighted.

To study the impact of pressure on the domain formation in nonferroelastic ferroelectrics, we utilize the hexagonal manganite system ErMnO_3_. Phase-pure ErMnO_3_ polycrystals are synthesized via a solid-state approach at a temperature of 1400 °C, which allows for obtaining dense samples with a cylindrical geometry (diameter ≈ 7.6 mm, height ≈ 5.1 mm; see methods and ref (27) for details). After synthesis, our polycrystals exhibit the typical *R*MnO_3_ domain structure as discussed in detail in ref (27). To study the impact of uniaxial pressure on the domain structure, we anneal our samples above the Curie temperature (*T*_c_ ≈ 1156 °C^28^) at 1220 °C and apply a constant uniaxial mechanical pressure along the long axis of the cylindrically shaped specimens while cooling to room temperature. A corresponding temperature–pressure profile is schematically displayed in Figure 1a. (See methods for a detailed description of the high-temperature annealing experiment.) We begin our discussion with one of the end cases, that is, an ErMnO_3_ polycrystal annealed under a maximum pressure of σ = 47 MPa reached in our experiment. The microstructure of this sample is analyzed via X-ray diffraction (XRD), electron backscattered diffraction (EBSD), and scanning electron microscopy (SEM) as summarized in Figure 1b. The XRD pattern reflects the hexagonal space group *P*6_3_*cm* without any indication of secondary phases. The pole figure in the inset to Figure 1b is evaluated over ∼400 grains, showing a nonpreferential crystallographic orientation of the grains.^29^ The SEM micrograph outlines an isotropic grain shape, indicating the absence of mechanically induced high-temperature microstructural creep.

Next, we image the ferroelectric domain structure by piezoresponse force microscopy (PFM) with a peak-to-peak voltage of 10 V applied to the back of the sample at a frequency of 40.13 kHz. To access the 3D distribution of the domains, we investigate different cross sections oriented perpendicular and parallel to the direction in which the mechanical pressure was applied during cooling. Corresponding images, showing the lateral PFM contrast, are displayed in Figure 1c,d, respectively. A pronounced PFM contrast is observed that allows for distinguishing the +*P* and −*P* domains. (See ref (27) for technical details.) For both cross sections, we predominantly find periodic patterns of stripe-like domains with a periodicity of about 50 nm. As highlighted by the white arrows, we consistently find a preferential orientation direction of the ferroelectric domain walls within one grain, which varies from grain to grain. Note that this behavior is completely different from ErMnO_3_ single crystals^19,30,31^ or polycrystalline samples cooled without mechanical pressure,^27^ where an isotropic vortex-like domain structure is predominant. The latter indicates a pronounced interaction between the domain formation and applied uniaxial pressure, which we investigate systematically in the following.

## Control of Domain Size and Orientation

To understand the relation between the applied mechanical pressure and the emergent domain structure, we perform annealing experiments under different mechanical pressures. To exclude pressure-induced changes of the crystal and microstructure, we first record XRD patterns and SEM micrographs of the samples. We find that the crystal structure of all samples can be described by the space group *P*6_3_*cm*, showing no indication of secondary phases (Figure S2). By analyzing about 20 grains in each sample, we measure an average grain size of 12.8 ± 1.7 μm (Figure S3). Importantly, our data shows that the grain size is independent of the applied pressure, discarding mechanically induced microstructural creep and the resulting grain-size effects as the origin of variations in the domain structure.^32^ Thus, going beyond previous grain-size-dependent studies,^27^ this sample series with grains of a single average size represents an ideal system for the investigation of pressure-driven phenomena.

**Figure 2:**
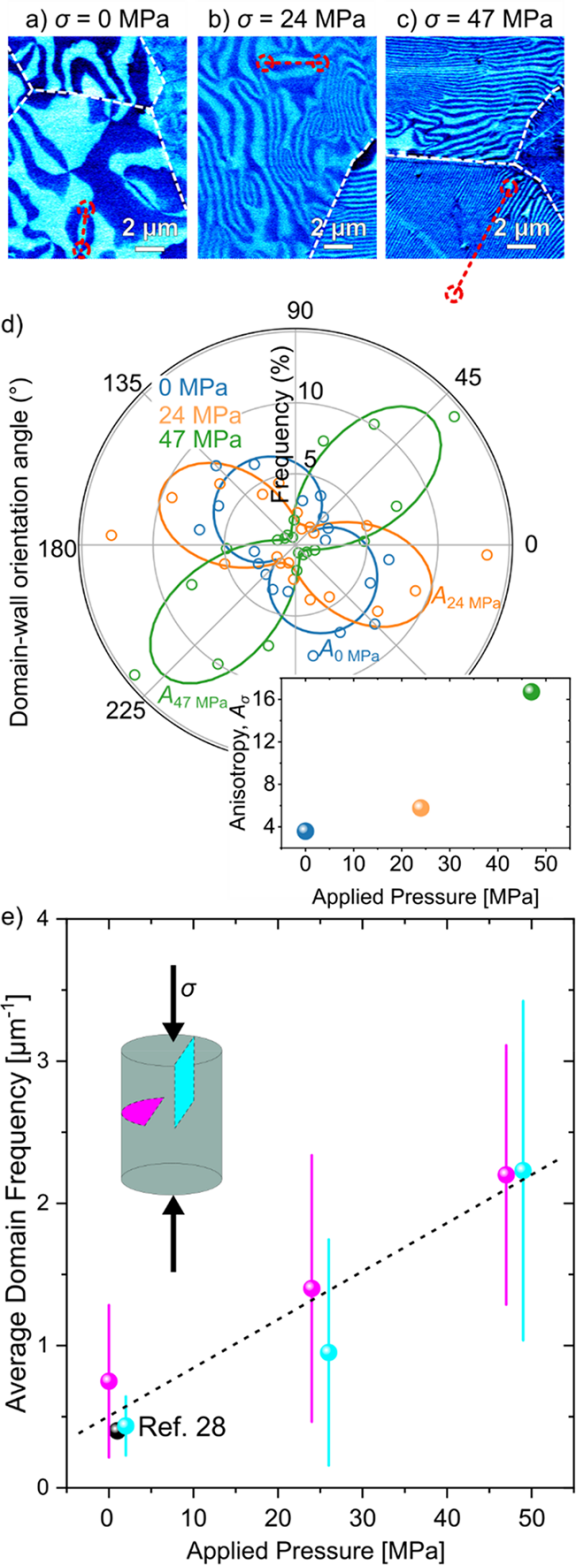
Control of the vortex density and domain frequency in polycrystalline ErMnO_3_ via mechanical pressure. Representative PFM images of polycrystalline ErMnO_3_ cooled under a) 0, b) 24, and c) 47 MPa. Overview images over an area of 50 × 50 μm^2^ of a section parallel and perpendicular to the mechanical pressure are displayed in Figure S4. Representative vortex/antivortex pairs are marked by dashed red circles in a) and b), whereas the respective antivortex core is outside the displayed area in c). A larger scanning area showing both the vortex and antivortex belonging to the pair is displayed in Figure S5. d) Relative distribution of the domain wall orientations displayed for different mechanical pressures for a representative grain. The orientation angle is measured relative to a reference line. The increasing degree of orientation of the stripe-like domains with increasing pressure is quantified by the anisotropy parameter *A*_*σ*_, quantified in the inset (a derivation of *A*_*σ*_ is provided in Figure S6; calculated uncertainties are displayed in Table S1). The average frequency of stripe-like domains is displayed as a function of the applied mechanical pressure in e). Cyan and purple data points represent the cross-section parallel and perpendicular to the applied mechanical pressure. A literature value (black data point) of the average domain frequency of single crystalline counterparts cooled under the same cooling rate (5 K/min) is displayed for comparison.^28^ The dashed line represents a linear fit to the experimental data. The error bars correspond to the standard deviations of the experimental data. Complementary statistical information is provided in Figure S7.

Representative PFM images of the domain structure in samples cooled under different mechanical pressures are displayed in Figure 2a–c. (Larger overview images covering 50 × 50 μm^2^ areas of parallel and perpendicular cross sections are displayed in Supporting Information Figure S3.) The domain structure displayed in Figure 2a (σ = 0 MPa) is consistent with previous observations,^27^ exhibiting a rather isotropic network of vortex domains within the different grains. The domain walls typically terminate at the grain boundaries and do not extend into adjacent grains, indicating that the ferroelectric domains in neighboring grains are largely independent as discussed in refs (27) and (33). Increasing the mechanical pressure during cooling to σ = 24 MPa transforms the isotropic vortex-like domain structure into a more stripe-like pattern (Figure 2b) in all grains, independent of their crystallographic orientation relative to the direction of the applied pressure. This stripe-like pattern reflects a preferred orientation for the domain walls within a single grain while varying in direction from grain to grain. The response of the domain structure in ErMnO_3_ to mechanical pressure is unexpected, since the domain walls are purely ferroelectric. The qualitative change in the domain structure thus cannot be explained based on the release of mechanical pressure via the formation of ferroelastic domain walls as in conventional ferroelectrics, such as BaTiO_3_, Pb(Zr,Ti)O_3_, and (K,Na)NbO_3_, pointing toward a different microscopic origin. As the comparison with PFM images acquired on samples annealed with a pressure of σ = 47 MPa shows (Figure 2c), the effect becomes even more pronounced as the mechanical pressure increases, leading to strongly elongated and highly ordered stripe-like domains within the different grains, as quantified by the pressure dependence of the anisotropy parameter *A*_*σ*_ obtained from the polar plots in Figure 2d (Figure S6). Note that the weak preferential orientation of the sample annealed in the absence of pressure is a consequence of the formation of stripe-like domains due to intergranular strain fields,^34^ a known feature for polycrystalline ErMnO_3_.^27^ The transition to a stripe-like domain structure naturally leads to larger distances between the 6-fold meeting points that form the characteristic vortex/antivortex pairs in hexagonal manganites, highlighted by the dashed circles in Figure 2a–c.

To quantify the mechanically driven change in the ferroelectric domain structure, we evaluate in addition to their directional ordering (Figure 2d) the domain frequency (Figure 2e). The domain frequency measures the number of domain intersections along the length of a test line drawn perpendicularly to the stripes, quantifying the size of the stripe-like domains.^35^ In Figure 2e, we present the domain frequency for a cross-section parallel (cyan) and perpendicular (purple) to the applied pressure by averaging over 40 grains for each direction. The data indicate a one-to-one correlation between the applied mechanical pressure and the frequency of the stripe-like domain structure, showing an enhancement in frequency by a factor of ∼4 as the mechanical pressure increases from 0 to 47 MPa. The same enhancement is observed in cross sections oriented parallel (cyan) and perpendicular (purple) to the applied pressure. The latter leads us to the conclusion that the orientation of the emergent stripe domains and associated domain walls is determined by the crystallographic orientation of the different grains rather than the direction of the applied uniaxial pressure, which we elaborate on in the following.

**Figure 3:**
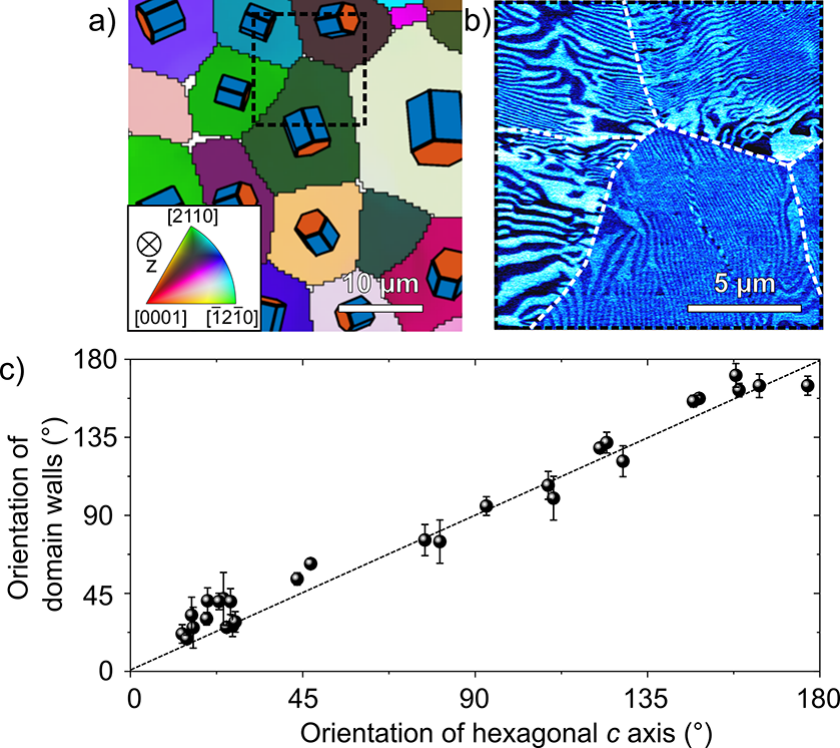
Control of the orientation of stripe-like domains. a) EBSD image of polycrystalline ErMnO_3_ thermomechanically treated under a pressure of 47 MPa (cross section perpendicular to the mechanical pressure) displaying the individual grains with their respective 3D direction as indicated schematically by the hexagonal prisms. An in-plane PFM image of the position marked with the black square is displayed in b). The grain boundaries are highlighted by dashed white lines. Comparing the EBSD and PFM images indicates that the orientation of the stripe-like domains is parallel to the *c* axis of the hexagonal prism. The correlation between the orientation of the stripe-like domains and the *c* axis of the hexagonal prism is displayed in c) and systematically analyzed for 29 grains with preferable in-plane orientation. The orientation of the domain walls associated with the stripe domains and the hexagonal *c* axis are measured with respect to the same reference line (details in Figure S8). The dashed black line indicates the ideal parallel orientation between the ferroelectric domain walls and the *c* axis of the prisms.

A more detailed analysis of the crystallographic orientation of the grains of our polycrystalline ErMnO_3_ (Figure 1c) in terms of spatially correlated EBSD and PFM measurements is presented in Figure 3 for a sample cooled under a mechanical pressure of σ = 47 MPa. EBSD measurements of a selected area are displayed in Figure 3a, where the orientation of the grains is schematically indicated by hexagonal prisms. A PFM image (in-plane contrast) of the area marked by the dashed black box in Figure 3a is given in Figure 3b. The data suggest that the stripe-like domains and associated domain walls predominantly align parallel to the crystallographic *c* axis (*P*||*c*). To corroborate this correlation, we analyze 29 grains and plot the domain wall orientation obtained from the PFM scans against the orientation of the *c* axis measured by EBSD in Figure 3c. (Orientations are measured with respect to the same reference plane, as explained in Figure S8.) Figure 3c confirms that there is a strong correlation between the pressure-induced stripe-like domain pattern and the crystallographic orientation of the individual grains, revealing a preference to align the walls parallel to the polar *c* axis when pressure is applied during annealing.

## Domain Structure

**Figure 4:**
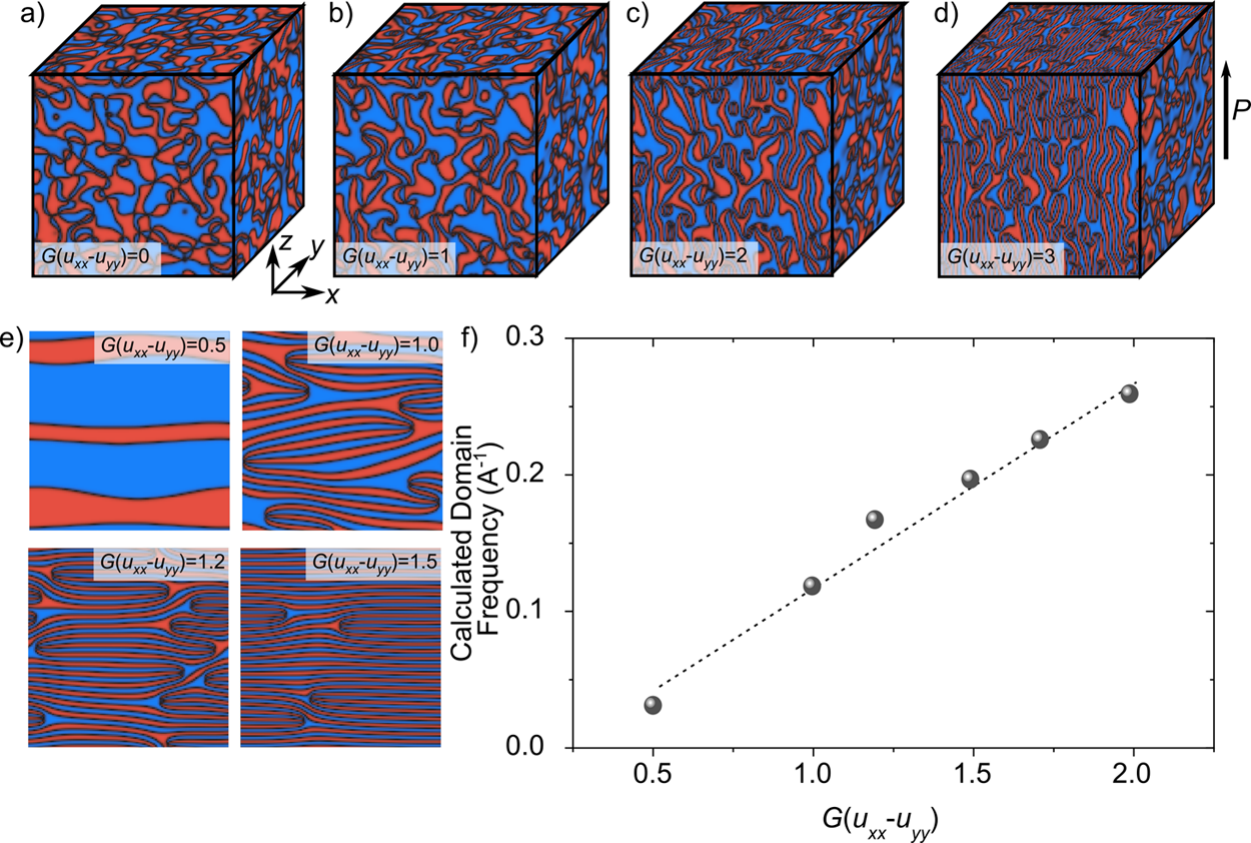
Phase-field simulations showing the relation between the mechanical pressure and the domain morphology in ErMnO_3_. The influence of the magnitude of applied pressure, *G*(*u*_*xx*_ – *u*_*yy*_), on the 3D domain structure is displayed in a)–d). To simulate a volume-preserving uniaxial pressure, a compression in the *x* direction is combined with a tension in the *y* direction. The ferroelectric polarization, *P*, is parallel/antiparallel to the *z* direction. The preferential formation of a layer-like domain structure with increasing magnitude of the pressure can be observed. Note that the domain structure within the *yz* plane, which displays a cut parallel to the individual layer-like structures, is not influenced by the mechanical pressure. The correlation between the applied pressure and the domain periodicity is displayed in e) and quantified in f). The dashed line represents a linear fit to the simulated data. Computational details are described in the Method Section.

To understand the pressure-induced changes in the domain structure and their relation to the crystallographic structure, we performed phase-field simulations. Following the established phase-field model for hexagonal manganites,^36,37^ we represent the system by two parameters, i.e., the trimerization amplitude *Q* and phase Φ as elaborated on in the Method Section. The model reproduces the characteristic vortex-like domain structure of ErMnO_3_ as displayed in Figure 4a, where the ferroelectric polarization is parallel/antiparallel to the *z* direction. The phase-field simulation allows for investigating the impact of uniaxial pressure within the volume, giving a 3D model of the pressure-induced domain structure. For this purpose, we consider the coupling between the energy density and the mechanical pressure,^36^
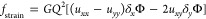
1where *G* is the strain coupling coefficient and (*x*, *y*) represents the Cartesian coordinates in the *xy* plane. Within the applied model (eq 1), the strain does not couple directly to the domain structure, which is fundamentally different from ferroelastic ferroelectrics.^38^ Instead, as described by eq 1, the elastic strain creates a force that pulls the vortices and antivortices away from each other.^26,37^ The difference between the strain components, *u*_*xx*_ – *u*_*yy*_, results in a modulation of the domain structure in the *x* direction, whereas the shear strain component, *u*_*xy*_, results in a modulation in the *y* direction. According to eq 1, the effect depends on the magnitude of the strain components and the respective phase gradients, *δ*_*x*_Φ and *δ*_*y*_Φ.

Our experimental parameter, that is, the mechanical pressure, is linked to the distribution of the individual strain components in the phase-field simulations, *u*_*xx*_, *u*_*yy*_, and *u*_*xy*_, via the anisotropic Young’s modulus.^39^ The 3D domain structure simulated for varying pressure amplitudes is displayed in Figure 4b–d). In agreement with our experimental findings (Figure 2a–c), the simulations reproduce a pressure-driven transition from an isotropic vortex-like (Figure 4a) to an anisotropic stripe-like (Figure 4d) domain structure. In comparison to previous two-dimensional simulations on ErMnO_3_,^27,36,37^ our three-dimensional phase-field model highlights that the stripe-like ferroelectric domains extend into an oriented layer-like structure in the third dimension. Consistent with the experimental data in Figure 3c, the simulations show that the pressure-induced stripe-like domains and associated walls preferentially orient parallel to the polar axis of the system. As expressed by eq 1, this is a consequence of the absence of the coupling of the energy density to the strain in the *z* direction, i.e., *u*_*zz*_. Physically, strains along the polar axis are invariant under all symmetry operations of the ErMnO_3_ system and hence merely result in a small shift of the Landau expansion parameters. Hence, these terms are usually ignored for Landau expansions in any order.^36^ Importantly, as expressed by eq 1, independent of the individual strain components, the stripe-like domains always orient preferentially parallel to the polar axis. While we can explain the experimentally observed emerging domain structures based on eq 1, additional electrostatic effects^40^ at the domain walls may further promote the formation of the stripe-like domain structure. In contrast to domain walls that are not parallel to *P*, the oriented stripe-like walls do not carry bound charges (div*P* = 0), which makes them energetically favorable. Importantly, based on the phase-field simulations, we can correlate the thickness of each stripe-like domain to the strain magnitude. 2D representations of the domain structure are displayed in Figure 4e, and the simulated domain frequency, *f*, is displayed as a function of the applied pressure, *G*(*u*_*xx*_ – *u*_*yy*_), in Figure 4f. We find that the domain frequency continuously increases with the applied pressure, which is consistent with the experimentally observed relationship (Figure 2e). As reported in ref (37), elastic strain modifies the vortex density via the creation and annihilation of vortex/antivortex pairs. Thus, the mechanical pressure provides a lever to transfer isotropic vortex-like domains into a periodic layered domain pattern with a tunable orientation and domain size.

A generally established concept to tune the dielectric and piezoelectric properties of ferroelectric materials and the backbone of their application as capacitors, sensors, and in energy storage devices is domain engineering.^1,41^ Conventionally, domain engineering is realized via the crystal structure or microstructure^1^ and the chemical composition^42^ of the materials or it is controlled via elastic strain.^12^ Thus, most existing concepts rely on the interaction between elastic strains and ferroelastic domains, which ultimately limits them to materials exhibiting ferroelasticity. Unlocked by elastic-strain-induced forces acting on the vortex/antivortex pairs,^36^ domain engineering was recently realized by utilizing elastic strain fields originating from confinement effects in nonferroelastic ErMnO_3_.^27^ Here, we expand this concept toward pressure-driven domain engineering, providing a conceptually new lever beyond microstructural effects. We find a one-to-one correlation between the applied pressure and the frequency and orientation of the induced stripe-like domains. Most importantly and different from ferroelastic ferroelectrics, such as BaTiO_3_, Pb(Zr,Ti)O_3_, and (K,Na)NbO_3_, our pressure-engineering approach does not require ferroelasticity. On the one hand, removing the need for ferroelastic domain walls expands the pool of candidate materials. On the other hand, it foreshadows a conceptually different method for mechanically switching ferroelectrics of an improper nature that does not result in unwanted changes in the physical shape of the sample due to the movement of ferroelastic domain walls.^43^ This would be beneficial to the lifetime of the ferroelectric material and crucial to its application in, e.g., memory devices or multilayer ceramic capacitors.

## Supporting Information Available

Method Section, providing additional experimental and theoretical details on the solid-state synthesis; thermomechanical treatment; structural and microstructural characterization; phase field simulations; overview PFM data; detailed crystallographic and microstructural analysis; and detailed determination of the orientation angle from PFM and EBSD data (PDF)
