# Supplementary material for: Pressure Control of Nonferroelastic Ferroelectric Domains in ErMnO3
Source: Nano Lett. 2023 Jul 20;23(15):6994–7000. doi: 10.1021/acs.nanolett.3c01638 (PMC10416345; doi:10.1021/acs.nanolett.3c01638)
Supplement: Supplementary file 1 — nl3c01638_si_001.pdf [file nl3c01638_si_001.pdf]

Supporting Information

Pressure Control of Nonferroelastic Ferroelectric Domains in $ErMnO_3$

Olav W. Sandvik[1], Aaron Merlin Müller[2], Håkon W. Ånes[1], Manuel Zahn[1,3], Jiali He[1], Manfred Fiebig[2], Thomas Lottermoser[2], Tadej Rojac[4], Dennis Meier[1,*], and Jan Schultheiß[1,3,*]

[1] Department of Materials Science and Engineering, Norwegian University of Science and Technology (NTNU), 7034 Trondheim, Norway

[2] Department of Materials, ETH Zurich, 8093 Zurich, Switzerland

[3] Experimental Physics V, University of Augsburg, 86159 Augsburg, Germany

[4] Electronic Ceramics Department, Jožef Stefan Institute, 1000 Ljubljana, Slovenia

*corresponding authors: dennis.meier@ntnu.no; jan.schultheiss@uni-a.de

1. Method Section

Solid state synthesis: Synthesis of $ErMnO_3$ powder was done by a solid-state reaction of dried Er_2O_3 (99.9% purity; Alfa Aesar, Haverhill, MA, USA) and Mn_2O_3 (99.0% purity; Sigma-Aldrich, St. Louis, MO, USA). The powders were mixed and ball milled (BML 5 witeg Labortechnik GmbH, Wertheim, Germany) for 12 hrs at 205 rpm using yttria stabilized zirconia milling balls of 5 mm and ethanol as dispersion medium. The reaction to $ErMnO_3$ was done by stepwise heating at 1000°C, 1050°C, and 1100°C for 12 hrs. More details on the powder processing can be found in ref. [1]. The powder was isostatically pressed into samples of cylindrical shape at a pressure of 200 MPa (Autoclave Engineers, Parker-Hannifin, Cleveland, OH, USA). Sintering was carried out in a closed alumina crucible with sacrificial powders of the same chemical compositions at a temperature of 1400°C for 4 hrs with a heating and cooling rate of 5°C/min (Entech Energiteknik AB; Ängelholm, Sweden).

Thermomechanical treatment: The cylinders with a diameter of ~7.6 mm and a height of ~5.1 mm were put into a Al_2O_3 die filled with coarse MgO powder to prevent the sample reacting with the die during heat treatment. The samples were heated to a temperature of 1220°C, thus above T_c of $ErMnO_3$ (T_c~1156°C [2]) with a heating rate of 5°C/min. After a dwell time of 30 minutes to thermally equilibrate the sample, a uniaxial pressure of either 0 MPa, 24 MPa, and 47 MPa was applied to the die, while maintaining a temperature of 1220°C for 10 more minutes. As illustrated in Figure 1a, the samples were cooled with the applied pressure until room temperature. For all experiments, a constant cooling rate of 5°C/min was utilized.

Structural and microstructural characterization: After thermomechanical treatment, the samples were cut into half-discs using a diamond wire saw (Serie 3000, Well, Mannheim, Germany) to get insight into the micro- and nanostructure parallel and perpendicular to the applied mechanical pressure. Next, the samples were lapped with a 9 µm-grained Al_2O_3 water suspension (Logitech Ltd., Glasgow, UK) and polished using silica slurry (Ultra-Sol® 2EX, Eminess Technologies, Scottsdale, AZ, USA). The crystal structure and phase purity of our samples were

determined by XRD (Bruker, Billerica, MA, USA). Micrographs of the polished samples were acquired by performing SEM (Helios G4 UX, FEI, Lausanne, Switzerland). The nanoscale domain structure of the samples was obtained via PFM (NT-MDT, Moscow, Russia) with an electrically conductive tip (Spark 150Pt, NuNano, Bristol, UK; cantilever stiffness of 18 N/m). The samples were excited using an alternating voltage with a frequency of 40.13 kHz and an amplitude of 10 V. The laser deflection was read out as the amplitude, R, and the phase, θ, of the piezoresponse by two lock-in amplifiers (SR830, Stanford Research Systems, Sunnyvale, CA, USA). Spatial resolution of the domain structure was obtained by displaying $R\cos\theta$, to qualitatively distinguish domains with different orientation. Prior to PFM measurement on the polycrystalline $ErMnO_3$, the PFM response was calibrated on a periodically out-of-plane poled $LiNbO_3$ sample (PFM03, NT-MDT, Moscow, Russia).

To map the orientation of the grains, EBSD was performed on an Ultra 55 FEG-SEM (Zeiss, Jena, Germany). To obtain sufficient statistics an area of 400 grains (approximately 235x235µm²) was mapped, whose domain structure was characterized by PFM prior to the EBSD analysis. Before the EBSD scan, the sample was carbon coated. The scan was performed with an acceleration voltage of 10 kV, a working distance of 20.4 mm and a sample tilt of 70°. Kikuchi diffraction patterns of 120x120 px² were recorded with a nominal step size of 0.5 µm. Diffraction patterns were indexed by dictionary indexing [3] followed by refinement as implemented in kikuchipy (version 0.8.0), [4] based on dynamical simulations of $ErMnO_3$ from EMsoft (version 5) [5]. Orientations used in dictionary indexing were sampled from the Rodrigues Fundamental Zone of proper point group 622. [6] Orientation analysis was performed in Matlab with MTEX (version 5.8). [7]

Phase-field simulations. We perform phase-field simulations based on the Landau expansion of the free energy $F(Q, \Phi, P, u_{xx}, u_{yy}, u_{xy})$. The Landau expansion is obtained by adding the ferroelectric and the strain terms of the Landau expansion. [8, 9] We choose the parameters of the ferroelectric term as in ref. [8], with the exception of $s_P^x = 8.88$ eV to ensure stability. The parameter G of the strain term is set as described in Figure 4 in the main text. For all simulations, we choose a uniform Cartesian, three-dimensional grid with lattice spacing $d_x = d_y = 0.2$ nm and $d_z = 0.3$ nm as computational mesh. We use periodic boundary conditions in the *x* and *y*-directions and open boundary conditions in the *z*-direction. We fix a uniform strain throughout the entire system and derive the Ginzburg-Landau equations for the structural and ferroelectric order parameters. [10] The Ginzburg-Landau equations are then integrated in a finite difference integration scheme using a Runge-Kutta 4 integrator with time step $\Delta t = 5 \cdot 10^{-4}$. The simulations of the three-dimensional crystals have been performed with a mesh of size $n_x \times n_y \times n_z = 128 \times 128 \times 128$. An initial, randomly generated field of all order parameters is evolved with uniform zero strain for $6 \cdot 10^{-4}$ time steps, such that a domain structure is formed. Then, the system is further integrated under uniform, constant strain for a time of $1.2 \cdot 10^5$ time steps.

To determine the stripe domain size, simulations with a mesh of size $n_x \times n_y \times n_z = 128 \times 128 \times 2$ are performed. Again, an initial, randomly generated field of all order parameters is evolved with uniform zero strain for $1.6 \cdot 10^5$ time steps to form an initial domain pattern. Then, the systems are integrated with uniform strain until the domains form a stripe-like pattern and no longer evolve. The integration time varies with the applied strain. Systems with lower strain evolve more slowly and therefore require more time steps. Finally, the stripe domain size is extracted using a stereological method. [11] Note that the calculated domain frequency (Figure 4f) is

smaller in comparison to the experimentally determined values (Figure 2e), which we relate to the fact that we set the parameter $G = 1$ in equation 1 for simplicity.

2. Structural and microstructural analysis

2.1.Piezoresponse Force Microscopy

Out-of-plane and in-plane Piezoresponse Force Microscopy (PFM) images of the same position as Figure 1c) and d) is displayed in Figures S1.

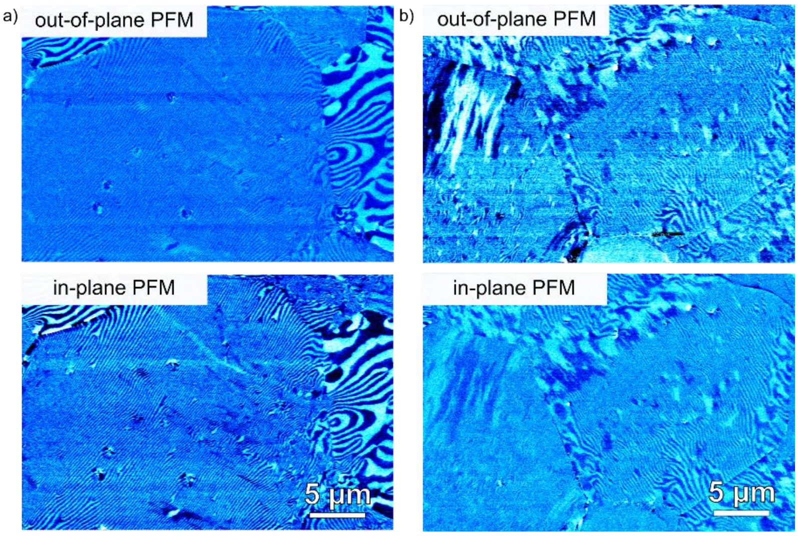

Figure S1: Out-of-plane and in-plane PFM images displaying the domain structure a) perpendicular and b) parallel to the applied mechanical pressure.

2.2.Crystallography

X-Ray diffraction (XRD) patterns of polycrystalline $ErMnO_3$ cooled under different mechanical pressures are displayed in Figure S2. The crystal structure of all samples can be described with the space group $P6_3cm$, while secondary phases are absent.

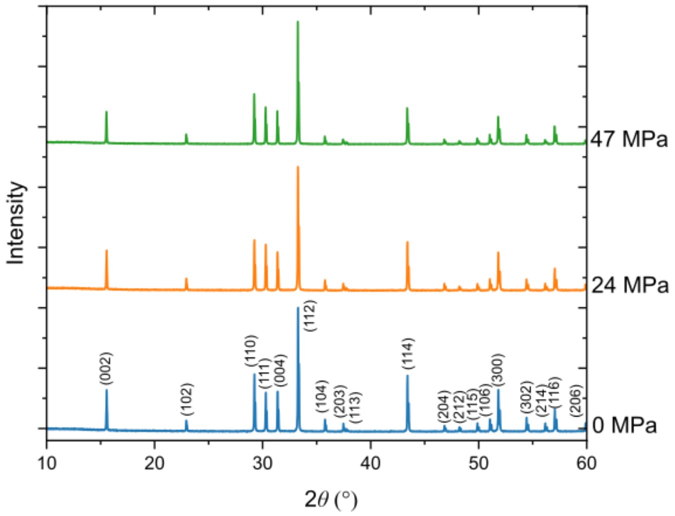

Figure S2: XRD pattern of polycrystalline $ErMnO_3$ cooled under different mechanical pressures.

2.3.Microstructure

Scanning electron microscopy (SEM) micrographs of polycrystalline $ErMnO_3$ cooled under different mechanical pressure are displayed in Figure S3. A systematic grain size analysis over 20 grains in each sample finds that the grain size is independent of the applied mechanical pressure and an average grain size of g=12.8±1.7µm was identified ($g_{0\ \mathrm{MPa}} = 13.3 \pm 1.4$ µm, $g_{24\ \mathrm{MPa}} = 10.9 \pm 1.4$ µm, and $g_{47\ \mathrm{MPa}} = 14.2 \pm 0.6$ µm). The microstructure features a mixture of inter- and intragranular microcracks, commonly found in polycrystalline hexagonal manganites, originating from a combination of a large volume change and a strong anisotropy in the thermal expansion coefficient of the hexagonal structure. [12, 13] In addition, ferroelectric domain and domain wall contrast can be observed in the SEM images, originating from electron emission yield as explained in ref. [14].

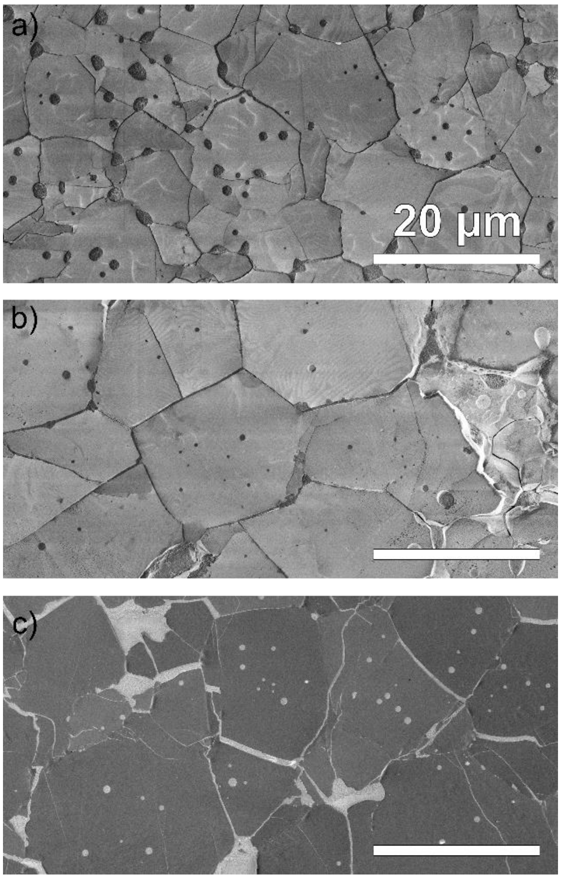

Figure S3: SEM images of polycrystalline $ErMnO_3$ cooled under different mechanical pressures. a) 0 MPa, b) 24 MPa, and c) 47 MPa.

3. Domain structure analysis via PFM

PFM images of a ferroelectric domain structure in polycrystalline $ErMnO_3$ cooled under different mechanical pressure, measured over an area of 50 x 50 μm^2 are displayed in Figure S4.

3.1.Overview PFM images

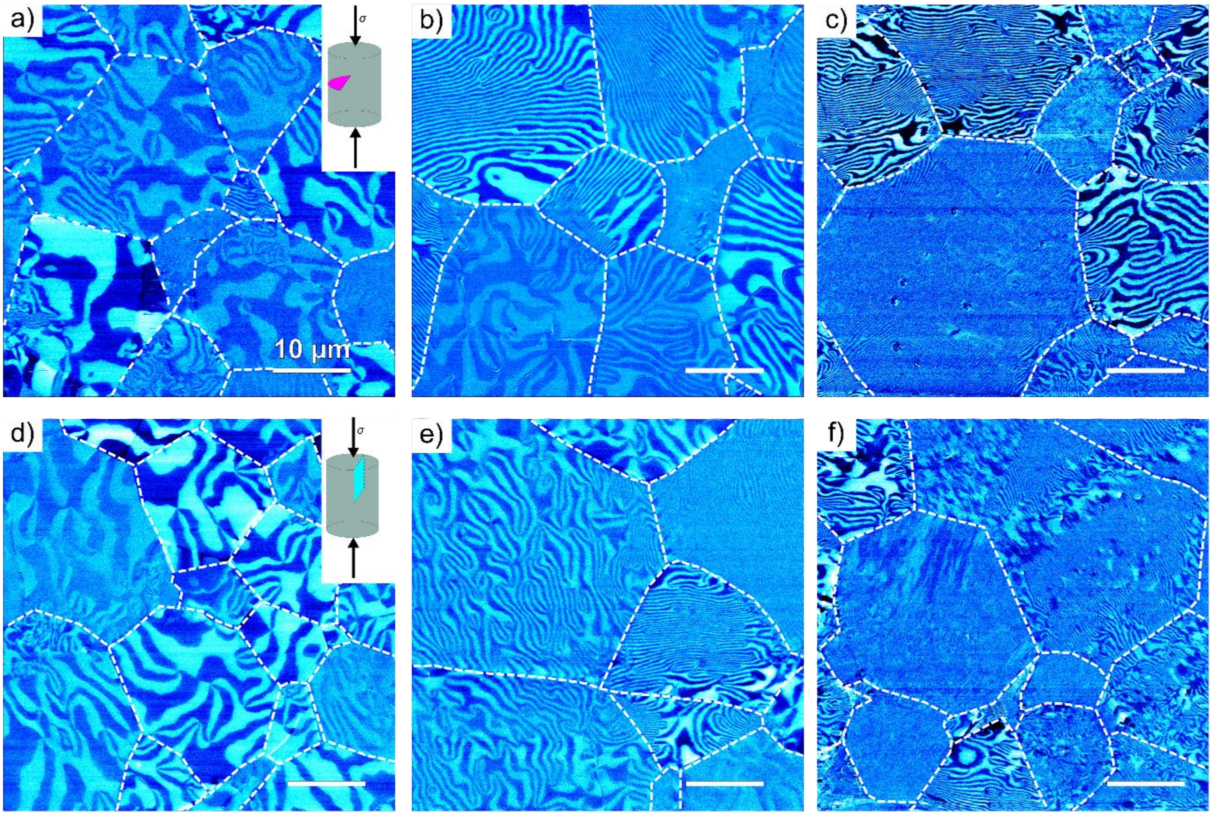

Figure S4: PFM images of polycrystalline $ErMnO_3$ samples cooled under different mechanical pressures. Images obtained from perpendicular and parallel cross sections are displayed in a)-c) and d)-f), respectively. The applied mechanical pressure is σ = 0 MPa (a) and d)), σ = 24 MPa (b), and e)) and σ =47 MPa (c) and f)).

3.2. Influence of mechanical pressure on domain structure

The mechanical-pressure driven domain formation leads to large distances between the sixfold meeting points, that form the characteristic vortex-/anti-vortex pairs in the hexagonal manganites. A representative example for the large distance is displayed in a PFM image in Figure S5, which is displayed as a complementary visualization to Figure 2c.

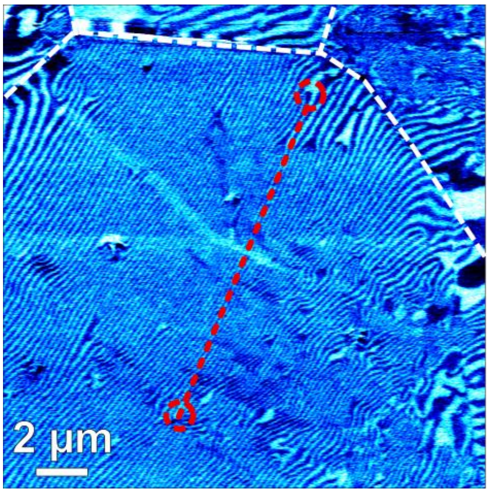

Figure S5: PFM image of polycrystalline $ErMnO_3$ cooled under a mechanical pressure of 47 MPa. The large distance between the vortex-/anti-vortex pairs in marked by dashed red circles.

The strategy to extract the domain wall orientations from individual grains for the PFM images is explained in Figures S6 (Figure 2d).

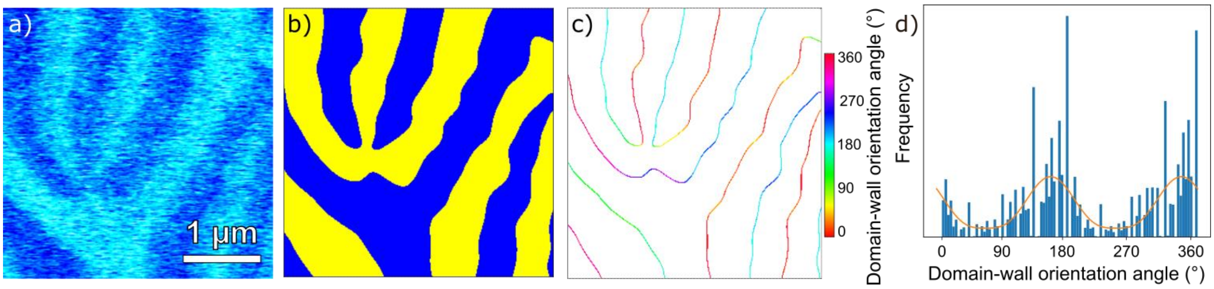

Figure S6: Extraction of domain wall orientations from PFM images, for an example of the sample annealed at σ=24 MPa. a) Section of the PFM phase image. First, a Gaussian filter is applied to reduce random noise. b) Via local thresholding using Otsu's method,[15] a binary image is obtained. Further, small islands of both colors with sizes much smaller than the characteristic length scale of the domains are removed. c) By applying Canny edge detection[16] and taking the local 2D derivative of the image, the position and local in-plane orientation of each domain wall segment is determined. d) Histogram of the in-plane domain wall orientation angles. Two peaks in opposite directions indicate the preferred domain wall orientation. The probability distribution is fitted to $\frac{1}{\kappa}\cdot\left(A_\sigma\cdot\left(\cos^2(\phi-\phi_0)\right)^w+1\right)$, where A_σ characterizes the height of the peaks compared to the flat regions, w the width of the peaks and ϕ_0 the preferred wall orientation. The parameter κ is the normalization constant, given by $\kappa = 2\pi + 4\cdot A_\sigma\cdot\ {}_2F_1(\frac{1}{2},\frac{1}{2}-w,\frac{3}{2},1)$, where ${}_2F_1$ is the hypergeometric function. The probability distribution is inspired by Malus' law that describes the transmittance of a polarizer and fulfills similar properties of symmetry.

The uncertainties on A_σ are calculated from the Cramér-Rao bound, using the Fisher-Information of the Likelihood function derived from the probability distribution (Table S1).

Table S1: Uncertainty, ΔA_σ, of the anisotropy parameter, A_σ.

Pressure [MPa]	A_σ	ΔA_σ
0	3.59	0.67
24	5.77	0.08
47	16.72	0.15

The data shown in Figure 2e is displayed as a box plot in Figure S7, providing insights into complementary statistical information to understand the role of the mechanical pressure on the frequency of the stripe-like domains.

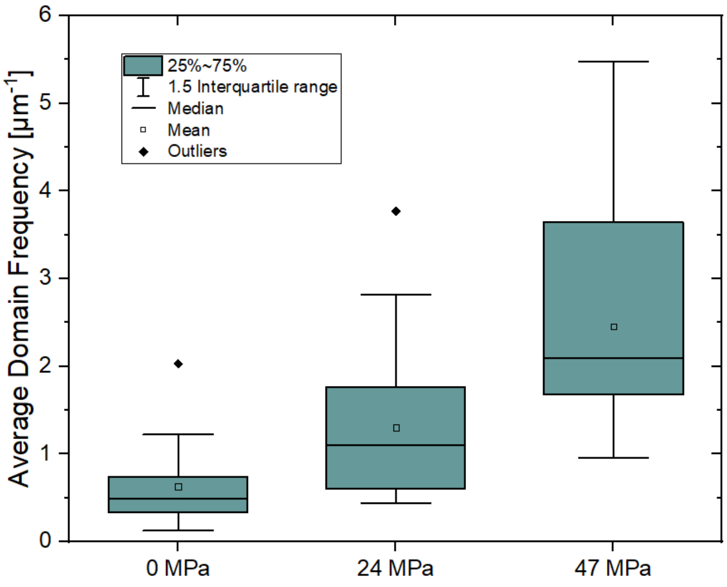

Figure S7: Frequency of stripe-like domains for samples cooled under different mechanical pressure (box plot of data displayed in Figure 2e), providing complementary statistical information on the influence of the mechanical pressure on the frequency of the stripe domains.

3.3.Control of the orientation of stripe-like domains

To understand the origin for the preferential orientation of the stripe-like domain walls, PFM, and electron backscattered diffraction (EBSD) data are collected on the same position. The orientation angle of the hexagonal c axis, α_{EBSD}, is quantified from EBSD data, while the orientation angle of the stripe domain walls, α_{PFM}, is obtained from PFM measurements. A horizontal reference line is utilized to quantify the angles and by aligning the EBSD and PFM images, a quantitative comparison, as done in Figure 3c becomes feasible. The quantification of these angles from the raw data is displayed for one grain in Figure S8.

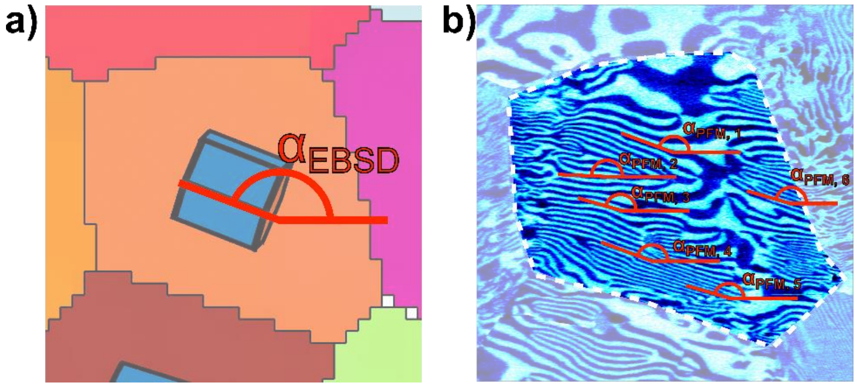

Figure S8: Quantification of the orientation angle of the a) hexagonal *c* axis from EBSD data, α_{EBSD}, and the b) orientation of the stripe domain walls from PFM data, α_{PFM}, sketched for one representative grain. To determine the orientation of the stripe domain walls, an averaged value from several domain walls is obtained.

References

[1] J. Schultheiß, F. Xue, E. Roede, H. W. Ånes, F. H. Danmo, S. M. Selbach, L.-Q. Chen, D. Meier, Pressure-control of non-ferroelastic ferroelectric domains in $ErMnO_3$, *Adv. Mater.* **2022**, *34*, 2203449.

[2] S. C. Chae, N. Lee, Y. Horibe, M. Tanimura, S. Mori, B. Gao, S. Carr, S. W. Cheong, Direct observation of the proliferation of ferroelectric loop domains and vortex-antivortex pairs, *Phys. Rev. Lett.* **2012**, *108*, 167603.

[3] Y. H. Chen, S. U. Park, D. Wei, G. Newstadt, M. A. Jackson, J. P. Simmons, M. De Graef, A. O. Hero, A Dictionary Approach to Electron Backscatter Diffraction Indexing, *Microsc. Microanal.* **2015**, *21*, 739.

[4] H.W. Ånes, L. Lervik, O. Natlandsmyr, T. Bergh, Z. Xu, E. Prestat, M. Nord, *pyxem/kikuchipy: kikuchipy 0.8.0* **2023**.

[5] P. G. Callahan, M. De Graef, Dynamical electron backscatter diffraction patterns. Part I: Pattern simulations, *Microsc. Microanal.* **2013**, *19*, 1255.

[6] S. Singh, M. De Graef, Orientation sampling for dictionary-based diffraction pattern indexing methods, *Model. Simul. Mater. Sci. Eng.* **2016**, *24*, 085013.

[7] F. Bachmann, R. Hielscher, H. Schaeben, Grain detection from 2D and 3D EBSD data—Specification of the MTEX algorithm, *Ultramicroscopy* **2011**, *111*, 1720.

[8] S. Artyukhin, K. T. Delaney, N. A. Spaldin, M. Mostovoy, Landau theory of topological defects in multiferroic hexagonal manganites, *Nat. Mater.* **2014**, *13*, 42.

[9] X. Shi, H. Huang, X. Wang, Phase-field simulation of strain-induced ferroelectric domain evolution in hexagonal manganites, *J. Alloys Compd.* **2017**, *719*, 455.

[10] L.-Q. Chen, Phase-field models for microstructure evolution, *Annu. Rev. Mater. Res.* **2002**, *32*, 113.

[11] E. E. Underwood, in *Quantitative Stereology for Microstructural Analysis*, (Ed: W. M. M. J. L. McCall), Springer US, Boston, MA **1973**.

[12] B. Fu, W. Huebner, M. F. Trubelja, V. S. Stubican, Synthesis and properties of strontium-doped yttrium manganite, *J. Mater. Res.* **1994**, *9*, 2645.

[13] C. Moure, J. F. Fernandez, M. Villegas, P. Duran, Non-ohmic behaviour and switching phenomena in $YMnO_3$-based ceramic materials, *J. Eur. Ceram. Soc.* **1999**, *19*, 131.

[14] K. Hunnestad, E. D. Roede, A. T. van Helvoort, D. Meier, Characterization of ferroelectric domain walls by scanning electron microscopy, *J. Appl. Phys.* **2020**, *128*, 191102.

[15] N. Otsu, A threshold selection method from gray-level histograms, *IEEE transactions on systems, man, and cybernetics* **1979**, *9*, 62.
[16] J. Canny, A computational approach to edge detection, *IEEE Transactions on pattern analysis and machine intelligence* **1986**, *6*, 679.
